# Correction: Spatio-temporal characterization of earthquake sequence parameters and forecasting of strong aftershocks in Xinjiang based on the ETAS model

**DOI:** 10.1371/journal.pone.0347626

**Published:** 2026-04-21

**Authors:** The PLOS One Editors

After publication of this article [1], concerns were raised regarding insufficient attribution of previously published methodologies described in [2] and [3], and about the completeness of reporting of the analyses and limitations.

The authors have provided further discussion of these issues upon editorial follow-up. In light of expert input received and the explanations and supporting information provided by the authors, the *PLOS One* Editors consider that the issues are resolved by this notice, which serves to provide appropriate attribution to the prior work in [2] and [3].

The following five sections summarize additional information and clarifications provided by the authors.

## 1. Additional details on the use of previously published methodology in [2] and [3]

The authors clarify that their analyses used tools and methodologies previously developed by another group [2] and [3] in their analysis of Xinjiang seismic data. The *PLOS One* Editors note that although the prior works were cited (as references 24 and 31 in [1]), the re-use of the code and partial re-use of text presenting Eqs. 1–10 and 16 in [1] were not clearly declared. Additionally, while [1] addresses a different research question and advances knowledge by applying the approach to the Xinjiang seismic data, similarities in the overall study design, methodology, and approach (including Figs 3 and 5) of [1] compared with the content (including Figs 2 and 4) of [3] were not discussed.

The authors acknowledge that they reused the three-part log-likelihood decomposition, iterative gradient-based approximation around the posterior mode, and the INLAbru for approximate Bayesian inference from [3], as well as the ETAS.inlabru methodology. In addition, they reused the overall workflow for fitting temporal ETAS models to earthquake catalogues from [2].

The authors further clarify that they newly developed or adapted the following elements: i) Application of the methodology to the Xinjiang earthquake catalogue (2009–2023); ii) LGCP-based spatial characterization of seismicity in Xinjiang (Eqs 12–15, Tables 2 and 3); iii) regional comparison of ETAS parameters across five seismic zones (Hotan, Yili, Kizilsut, Bortala and Kashgar) with ranking of aftershock triggering ability (α) and decay rate (p), and comparison with Chinese mainland averages; iv) time-varying parameter analysis with cutoff dates [1, 2, …, 30] days after the 2014 Hotan Ms7.3 mainshock (Fig 2); and v) MCMC (Bayesian ETAS) vs INLA (InLabru) comparison on the Xinjiang dataset, including goodness-of-fit and synthetic catalogue comparison.

The authors acknowledge that Eqs. 1–10 in the Research models and methods section closely follow [3]. Specifically, Eq. 1 in [1] corresponds to Eq. (3) in [3] (ETAS conditional density); Eq. 2 in [1] to Eq. (5)/Table 1 in [3] (Hawkes intensity); Eq. 3 in [1] to Eq. (9) in [3] (log‑likelihood); Eq. 4 in [1] to Eqs. (11)-(13) in [3] (Λ decomposition); Eqs. 5–6 in [1] to Eq. (14) in [3] (time‑bin structure and three‑part formulation); and Eqs. 7–10 in [1] to Section 4 in [3] (linear approximation, Poisson surrogate, and parameter transformation).

The authors report that the following package versions were used in the analyses: InLabru (v 2.13.0), INLA (v 24.6.27), and bayesianETAS (v 1.0.3). Their custom implementation adapts the methodological framework established in [3], and the ETAS.inlabru package. All software packages were used as provided, without modification.

The code used for the methodology and for generating the figures in prior works [2,3] is available at https://edinburgh-seismicity-hub.github.io/ETAS.inlabru/ and https://github.com/Serra314/Hawkes_process_tutorials/blob/main/how_to_use_Hawkes/report_ETAS.2022-10-04.pdf.

## 2. Reporting of parameter robustness

The authors provide the following additional information:

A prior sensitivity analysis was conducted of the key parameters a and p in the ETAS model for the Ms7.3 sequence in Hotan region. Based on the comprehensive consideration of seismological background knowledge, statistical rationality and numerical stability a gamma prior was selected for Bayesian modeling of multiple model parameters (μ, K, α, c, p). The gamma distribution only supports positive values, naturally conforms to the non-negative constraint of ETAS parameters (p > 1), and is convenient for the numerical stability of the INLA algorithm. The specific parameter priori settings are as follows μ ~ Gamma (shape = γ, rate = 10γ), K ~ Gamma (shape = 2γ, rate = γ), α ~ Gamma (shape = 2γ, rate = γ), c ~ Gamma (shape = γ, rate = 10γ), p ~ 1 + Gamma (shape = γ, rate = 5γ), and super parameter γ value set to 28, 29, 30, 31, respectively. As shown in the sensitivity analysis in S1 Fig, under different γ values, the posterior distribution of parameters α and p converges to a similar region, and the posterior results remain stable, supporting that the method has low sensitivity to the degree of prior dispersion. An a priori predictive check of parameters was conducted using Hotan Ms7.3 as an example; as shown in S2 Fig, the real data falls in the peak area of the simulated distribution, indicating that the priori setting selected is reasonable.

## 3. Clarification of the linkage between LGCP and ETAS

The authors provide the following additional information:

The core objective of the LGCP model is to characterize the spatial distribution characteristics of earthquakes in Xinjiang from 2009 to 2023, estimate the range (mean 1.995), standard deviation (mean 1.121) and other parameters of the Gaussian random field through the INLA algorithm, verify the spatial clustering of earthquakes in Xinjiang (the confidence interval of the standard deviation does not contain zero), and identify five high-risk regions of earthquakes in Hotan, Kashgar, Kizilsu, Ili and Bortala based on the posterior mean ranking of the spatial random fields. Because the geological structure of different regions in Xinjiang is significantly different, we first divide high-risk regions through LGCP, and then fit the ETAS model for each region separately to more accurately capture the region specific aftershock activity law. However, although this paper considers the use of LGCP model to clarify that Xinjiang earthquakes have spatial clustering differences (such as the difference of posterior mean between Hotan and Altay regions), the background rate (μ) of ETAS is only estimated based on the overall time series data of a single region, and does not combine the spatial parameters of LGCP with the background rate (μ) of ETAS for detailed research.

In the ETAS model built in this study, the output results of the LGCP model are not used to build the background seismicity rate μ with spatial variation characteristics.

## 4. Sensitivity to the completeness magnitude Mc = 3.0

The authors provide the following additional information:

To evaluate the impact of Mc uncertainty on ETAS key parameters α and p, multi-Mc value comparison experiments were conducted for two typical regions, Hotan Ms7.3 sequence and Kashgar Ms6.4 sequence. As shown in S3 andS4 Figs and S1-S2 Tables, the key parameters α and p are weakly affected by the Mc estimates. The conclusions of this paper based on the parameters α and p of Mc = 3.0 are statistically robust, and the estimation error of Mc does not change the qualitative judgment of the earthquake sequence.

The value range of Mc covers the uncertainty of the integrity magnitude estimated by the MAXC method, reflecting the possible fluctuation of the magnitude integrity in the actual earthquake catalog. Through sensitivity analysis within this interval, the robustness of ETAS parameters (especially α and p) to Mc selection can be evaluated.

## 5. Consistency between INLA and MCMC implementations

The authors provide the following additional discussion of limitations:

A limitation of this study is that a direct comparison between INLA and MCMC on the posterior marginal distribution of ETAS model parameters (μ, K, α, c, p) was not conducted. There is a strong correlation between ETAS model parameters (such as α and p), and INLA, as an approximate inference method, may have deviation when dealing with such strongly correlated posterior structures. A quantitative assessment of this could potentially strengthen the demonstration of consistency between INLA and MCMC methods, but was beyond the scope of this study. However, [2] and [3] directly evaluated the consistency between INLA‑based (inlabru) and MCMC‑based (bayesianETAS) approaches for ETAS‑type Hawkes models and reported that the two techniques produce similar parameter estimates. This prior validation supports that the INLAbru and MCMC fits are nearly identical in the present study (Fig 3), with INLA requiring substantially less computational time (0.30 min vs. 1.48 min for the Hotan Ms 7.3 sequence). It further supports the study’s conclusion that the INLA approach can more accurately forecast earthquake numbers during periods of sudden activity (Fig 6).

## Supporting information

S1 Fig The posterior distribution of parameters α and p under different γ values. (DOCX)

S2 Fig Priori predictive check for Hotan Ms7.3 and the priori setting results as shown in Fig. S1. (DOCX)

S3 Fig Posterior distributions of etas parameters α and p for the Hotan Ms7.3 sequence under varying integrity magnitudes (Mc) values. (DOCX)

S4 Fig Posterior distributions of etas parameters α and p for the Kashgar Ms6.4 sequence under varying integrity magnitudes (Mc) values. (DOCX)

S1 Table The posterior mean and 95% confidence interval of parameters α and p in Hotan area under different integrity magnitudes (Mc). (DOCX)

S2 Table The posterior mean and 95% confidence interval of parameters α and p in Kashgar area under different integrity magnitudes (Mc). (DOCX)
