# Supplementary material for: Spatio-temporal characterization of earthquake sequence parameters and forecasting of strong aftershocks in Xinjiang based on the ETAS model
Source: PLoS One. 2024 May 16;19(5):e0301975. doi: 10.1371/journal.pone.0301975 (PMC11098309; doi:10.1371/journal.pone.0301975)
Supplement: Attachment — Submitted filename: Response.docx [file pone.0301975.s001.docx]

**Original Manuscript ID:** PONE-D-23-37311

**Original Article Title:** “Spatio-temporal characterization of seismic sequence parameters and prediction of strong aftershocks in Xinjiang based on the ETAS model ”

**To:** Academic Editor

**Re:** Response to reviewers

Dear Editor,

Thank you for allowing a resubmission of our manuscript, with an opportunity to address the reviewers’ comments.

We are uploading (a) our point-by-point response to the comments (below) (“Response*”*), (b) an updated manuscript with yellow highlighting indicating changes(“Highlighted*”*), and (c) a clean updated manuscript without highlights (“Main Manuscript”*).*

Best regards,

<Xijian Hu> et al.

**Reviewer#1, Concern # 1 :**

How you could show that the results are reliable?

**Author response:** Thank you for your valuable questions! First of all, we ensure the reliability of the data source of the research object, taking the seismic events in the "National Earthquake Cataloging System" provided by China Earthquake Networks Center from January 1, 2009 to July 2, 2023 as the research object, and we also add the website of the data source in the paper. Secondly, we conduct completeness analysis of the seismic catalog to prevent too high a value resulting in under-sampling and the loss of available data, and to prevent too low a value resulting in incorrect seismic activity parameter values, resulting in biased analysis using incomplete data. Finally, the goodness of fit test of the model is carried out in this paper, and the test results show that the sequence values generated by the INLAbru and MCMC methods when fitting the ETAS model are very close to the actual true values, indicating that the model results obtained by the two methods can well reflect the data in this region.

**Author action:** We have added the detailed data sources in the paper, the completeness analysis of the seismic catalog in the "Appendix" of the document, and the model fitting degree test included in the paper can well illustrate the reliability of the results in the paper.

**Reviewer#1, Concern # 2:**

More analysis are needed to show that variation of results when the parameters change.

**Author response:** Thank you very much for your valuable advice! We added more analyses to explain the change of the results when the parameters changed: Both the α value and the p value of the ETAS model parameters showed a sudden change 15 days after the earthquake, especially the α value of the ability to trigger secondary aftershocks had the most drastic change, and the p value fluctuated to a certain extent within 13 days after the earthquake. The reason for the "sudden" change of sequence parameters can be simply analyzed from the seismic sequence activities, which may be because a large magnitude aftershock occurred in the early stage after the main earthquake, so the seismic sequence has a large change in the decay rates of aftershock and the excitation degree of aftershock.

**Author action:** We have added a detailed analysis of "Time-varying characteristics of seismic sequence parameters" in the article.

**Reviewer#1, Concern # 3 :**

There some new works on EATAS model which should be cited in the text:
-Temporal clustering PSHA based on smoothing: a case study in the New Madrid seismic zone of the central USA
-Probabilistic Seismic‐Hazard Analysis Including Earthquake Clusters

**Author response:** Thanks for your suggestions! We have added these two research results as references in the introduction part of this paper.

**Author action:** These two studies have been added to the references of this paper. For details, see references [14] and [15].

**Reviewer#1, Concern # 4 :**

- limitations of analysis should be put in the text.

**Author response:** Thank you very much for your advice! We have carefully considered the limitations and shortcomings of the study in this paper. Due to the limitations of the number of strong earthquakes in the study area and the computational conditions of the ETAS model, we only estimated the seismic sequences in the five high seismicity areas in Xinjiang, and discussed the temporal characteristics, and the conclusions drawn only reflect some of the statistical characteristics, and we will need to continue to accumulate more examples for more in-depth study.

**Author action:** We have taken your valuable comments and included the limitations of this study in the final conclusion of the article.

**Reviewer#2, Concern # 1 :**

-The authors do not demonstrate a compelling case for determining the magnitude of completeness quoted, or to show that it does not vary in time during the sequences. In particular, it is well known then threshold goes up early in the sequence when there are a lot of events with overlapping seismograms, so the smaller events are hidden in the wave-train of the larger events (Peng et al., 2007). The authors should show the method of estimating completeness explicitly instead of just citing a paper describing the method used then giving the result. This will allow the reader to make an independent judgement on the choice, and is needed to demonstrate (or not) that there is no change in the completeness threshold early in the aftershock sequences. This could be illustarted by adding a ‘dot plot’ of event magnitude v. time for example.

**Author response:** Thank you very much for your reminding! We have explicitly described a method for estimating the completeness magnitude of earthquakes. In this article, we have used the Maximum Curvature method (MAXC), which is a fast and direct method for estimating the completeness magnitude of an earthquake catalog. It is defined by calculating the maximum value of the first derivative of the cumulative magnitude frequency distribution curve, which usually matches the magnitude corresponding to the most frequent event in the non-cumulative magnitude frequency distribution. From the cumulative frequency distribution (Cum.FMD) and non-cumulative frequency distribution (Non Cum.FMD) of the Xinjiang earthquake catalog provided in Figure 2, it can be directly seen that the earthquake completeness magnitude is 3.0.

**Author action:** In the "Research Data" section of the paper, we have added the completeness analysis of the earthquake catalog, and used the maximum curvature method (MAXC) to obtain the completeness magnitude of the earthquake catalog in Xinjiang region during this period.

**Reviewer#2, Concern # 2 :**

-The authors use the term ‘prediction’ or ‘predicted’ several times in the manuscript. This is a bit of a misleading term because it implies a degree of determinism that is at odds with a stochastic model for a complex system. I recommend the term ‘forecasting’ is used instead, consistent with the wider literature on predictability (including other applications such as weather forecasting), and the operational use of the methods for risk communication during seismic sequences (Jordan et al, 2011) which could also be quoted as a motivation for this study.

**Author response:** Thank you very much for your advice! We have taken your suggestion and replaced the word "predict" or "predicted" with the term "forecasting" wherever it appears in the text.

**Author action:** We have corrected all misleading words in the text based on the reviewers' comments.

**Reviewer#2, Concern # 3 :**

-In setting the context for the paper the authors should cite recent work on earthquake forecasting using INLAbru by Bayliss et al (2020, 2022) and Naylor et al (2023) which precedes the work reported here. The paper also needs an introduction to R-INLAbru itself, and a url so readers could access it and check the results presented here independently. It would also be useful to know what are the similarities and differences of the current method with respect to this earlier body of work. Are there are novel aspects of the methods used, or is the main novelty in applying these to a different tectonic area?

**Author response:** Thank you very much for your reminding! We have added references to the work of Bayliss et al. (2020, 2022) and Naylor et al. (2023) in the introduction section of the article. We have taken your valuable advice and added a brief introduction to the R-INLAbru package and provided its URL.

Compared with the previous work, the novel feature of our study is to apply this method to seismic sequences in different regions of Xinjiang, and study the spatio-temporal characteristics of seismic sequence parameters and the forecasting of strong aftershocks in these regions. At the same time, our research results show that ETAS model can effectively forecasting the number of early seismic events after the occurrence of strong aftershocks, and it is found that INLA algorithm can forecast the sudden period of earthquake events more accurately than MCMC algorithm, which is more conducive to the research of strong aftershock forecasting.

**Author action:** We have added references [6], [7] and [24] to the introduction section of the text, and added a brief description and URL of INLAbru package to the "Epidemic Type Aftershock Sequence (ETAS) Modeling" section of the article.

**Reviewer#2, Concern # 4 :**

-The standard of proof reading of the manuscript is quite variable. I could not find all of the cited references, there are many phrases hanging from what looks like a previous edit, and many statements that I could not understand or that could have more than one or a misleading meaning. I would recommend the senior author carries out a careful proof read of the presentation in English before any resubmission.

**Author response:** We apologize profusely for our careless mistake and thank you for your reminder!

**Author action:** We have systematically solved the problems in the reference part of the article and added relevant references according to your comments, and modified the language expression of the full text sentence by sentence according to your suggestions, trying to achieve a clear expression of the article.

**Reviewer#2, Concern # 5 :**

-Table 1 should also include the quantiles for each parameter. This would allow the comparison of parameters on the next page to be made more definitive – the parameters would either be within the uncertainty range of each other or not. Some of the claimed differences may not be significant at this level, so it is important to concentrate on those that are.

**Author response:** Thank you very much for your valuable advice! We have taken and added the quantiles for each parameter of the model in Table 1 of the paper.

**Author action:** We have taken the reviewer's comments and added the quantiles for each parameter in Table 1.

**Reviewer#2, Concern # 6 :**

I would recommend adding the 95% confidence limits for the counting errors, conditional on the forecast number of events N (i.e. $\pm$1.96N), to show the forecast uncertainty is similar to or a bit wider than the ideal case where the number is forecast accurately (if not precisely).

**Author response:** Thank you very much for your valuable advice! We have adopted to increase the confidence interval of the counting error to 95%. The results show that it is indeed closer to an accurate forecast.

**Author action:** We have taken the reviewer's comments and made changes to increase the confidence interval for the counting error to 95%.

**Reviewer#2, Concern # 7 :**

The conclusion takes the form of an extended abstract and is too long. It should be rewritten to present the main findings and discoveries of the work and its wider significance.

**Author response:** Thank you very much for your valuable advice! We have taken your suggestion to provide a focused brief overview of the concluding section at the end of the article.

**Author action:** We have made changes to the concluding section at the end of the text.
