# Supplementary material for: Spatio-temporal characterization of earthquake sequence parameters and forecasting of strong aftershocks in Xinjiang based on the ETAS model
Source: PLoS One. 2024 May 16;19(5):e0301975. doi: 10.1371/journal.pone.0301975 (PMC11098309; doi:10.1371/journal.pone.0301975)
Supplement: Attachment — Submitted filename: Response to Reviewers.docx [file pone.0301975.s002.docx]

**Original Manuscript ID:** PONE-D-23-41552R1

**Original Article Title:** “Spatio-temporal characterization of earthquake sequence parameters and forecasting of strong aftershocks in Xinjiang based on the ETAS model ”

**To:** Academic Editor

**Re:** Response to reviewers

Dear Editor,

We feel great thanks for your professional review work on our article. As you are concerned, there are several problems that need to be addressed. According to your nice suggestions, we have made extensive corrections to our previous draft, the detailed corrections are listed below.

We are uploading (a) our point-by-point response to the comments (below) (“Response to Reviewers *”*), (b) an updated manuscript with yellow highlighting indicating changes(“Revised Manuscript with Track Changes*”*), and (c) a clean updated manuscript without highlights (“Manuscript”*).*

Best regards,

<Xijian Hu> et al.

**Academic Editor, Concern # 1 :**

**Author response:** We deleted Figure 1 and Figure 3 in the manuscript and replaced the necessary content in Figure 3 with Table 3 to describe it. Table 3 lists the posterior results of spatial random fields fitted according to LGCP model in 10 regions of Xinjiang.

**Reviewer#2, GENERAL COMMENTS, Concern#1 :**

-It would be very convenient for our conversation here, if the lines were numbered

**Author response:** Thank you very much for your reminder! We have numbered each line in the manuscript, to facilitate better communication with you.

**Author action:** We have numbered each line in the manuscript, to facilitate better communication with you.

**Reviewer#2, GENERAL COMMENTS, Concern#2:**

-In many places throughout the text, some sentences are too long to clearly express and describe either the content or the scope of what is described to be done.

**Author response:** We sincerely thank you for your careful reading. Based on your suggestions, we have made revisions to the parts of the manuscript that have too long sentences and vague descriptions.

**Author action:** We have made revisions to the manuscript for long sentences and vague descriptions. The location of the revised parts is as follows: Page 1, line 30; Page 2, line 56; Page 2, line 79；Page 2, line 88；Page 3, line 108；Page 4, line 145；Page 6, line 222；Page 7, line 252；Page 9, line 303；Page 10, line 329；Page 13, line 445 (Second,...)；Page 15, line 469. We have highlighted all the modifications.

**Reviewer#2, GENERAL COMMENTS, Concern#3 :**

- A careful reading will improve grammar and syntax – it is necessary.

**Author response:** Thank you very much for your advice! We have sought the help of English language professionals and have re-examined the manuscript in detail, revising the sections with grammatical and syntactical problems.

**Author action:** We have made the following modifications to the parts of the text that have grammatical and syntactical problems. The location of the revised parts is as follows: Page 1, line 16；Page 1, line 18; Page 1, line 26; Page 1, line 37; Page 2, line 50; Page 3, line 101; Page 3, line 131; Page 8, line 297; Page 11, line 365; Page 14, line 461; Page 16, line 494; We have highlighted all the modifications.

**Reviewer#2, GENERAL COMMENTS, Concern#4 :**

-Please, say earthquakes – why are seismic events, or simply events?

**Author response:** Thank you for your careful examination. Based on your suggestions, we have revised the words "seismic events" "events" in the text to "earthquakes".

**Author action:** We have checked the manuscript and revised the words "seismic events" "events" in the text to "earthquakes". The location of the revised parts is as follows: Page 1, first paragraph, line26；Page 1, first paragraph, line 27；Page 2, line 43, etc. We have highlighted all the modifications.

**Reviewer#2, MAJOR COMMENTS, Concern # 1:**

-Page 12, 1st paragraph: The difference of parameters values among the sequences must be discussed and interpreted appropriately.

**Author response:** Thank you very much for your suggestion! Based on your suggestion, we have added a discussion and explanation of the differences between the sequence parameters to the manuscript. The details are as follows: Overall, there were differences in the degree of variation from the sequence parameter values, with the minimum degree of variation in the parameter c and K values, while the degree of variation in the parameter mu, p, and alpha values was very obvious. The reason for the above result is that the parameter c is a very small positive constant, which is mainly considered to include a small constant term in the model equation to avoid a zero denominator. It therefore does not have a very clear physical meaning. It can also be seen from Eq. (2) that the parameters p and alpha are in exponential positions in the model expression, and thus c has less influence compared to p and alpha. Therefore, among the parameters of the ETAS model, the variation of c over time is relatively smooth, while the variation of alpha and p is more obvious.

**Author action:** We have added a discussion and explanation of the differences between the sequence parameters to the manuscript. The location of the revised parts is as follows: Page 11, line 384.

**Reviewer#2, MAJOR COMMENTS, Concern # 2 :**

-Page 13, 1st paragraph, 2nd line: “almost the same” are in all similar cases of the published results but there is extensive discussion on the differences as far as the time they occur and their magnitude. Please, discuss and interpret these differences.

**Author response:** We think this is an excellent suggestion. We have added Fig 4 ("Time-magnitude plots of earthquake sequences generated by the ETAS model and actual earthquake sequences are fitted based on the INLAbru, MCMC method.") to the manuscript, which shows the differences in the occurrence times and magnitude sizes of the earthquake sequences generated by the two methods of fitting the ETAS model, MCMC and INLAbru, and the actual earthquake sequences. Based on Fig 4, we are able to conclude that INLAbru generates earthquake sequences with occurrence times and magnitude sizes closer to actual earthquake sequences compared to the MCMC method.

**Author action:** We have added a comparison of the occurrence times and magnitude sizes of the earthquake sequences generated by fitting the ETAS model with the actual earthquake sequences by the two methods MCMC and INLAbru to the manuscript. The location of the revised parts is as follows: Page 13, line 438；Page 18, line 541.

**Reviewer#2, SPECIFIC COMMENTS, Concern # 1 :**

-Page 11, 2nd paragraph: “the form of tectonic movements” – do you mean the type of faulting? Or the deformation rates? Please be specific.

**Author response:** Thank you very much for your suggestion! We have revised this part as you suggest by changing "the form of tectonic movements" to "different types of faults in each region".

**Author action:** We have made modifications to this part based on your suggestions. The location of the revised parts is as follows: Page 11, line 339.

**Reviewer#2, SPECIFIC COMMENTS, Concern # 2 :**

-Page 11, 2nd paragraph: “ … the different types” – same as before. please, clarify what do you mean by “different types of seismic ruptures”

**Author response:** Thank you very much for your suggestion! We have revised this part as you suggest by changing "… the different types" to "different sequence types of aftershocks".

**Author action:** We have made modifications to this part based on your suggestions. The location of the revised parts is as follows: Page 10, line 338.

**Reviewer#2, SPECIFIC COMMENTS, Concern # 3 :**

-Page 11, 2nd paragraph: “ … we can observe … in alpha values” – You need to compare the a – values with the ones obtained in other regions worldwide

**Author response:** We sincerely appreciate your valuable suggestions! Based on your suggestion, we have added this part to the manuscript to compare our calculated alpha values with the alpha values of the moderate to strong earthquake sequences in mainland China (the distribution of the mean range of alpha is obtained from Ref [37]) .

**Author action:** We have taken your suggestion to add this part to the manuscript and have added the Ref [37]. The location of the revised parts is as follows: Page 10, line 349.

**Reviewer#2, SPECIFIC COMMENTS, Concern # 4 :**

-Page 11, 2nd paragraph: “ … in p values” – Could you interpret the p – values in connection with physical and mechanical characteristics of the activated area? There are plenty relevant publications on that aspect

**Author response:** We are very glad for your suggestion! Based on your suggestion, we have added the physical meaning of the p-value to the manuscript, which characterizes the ability of an earthquake sequence to decay, with a large p-value resulting in a rapid decay of the sequence and a small p-value resulting in a slow decay of the sequence.

**Author action:** We have taken your suggestion to add this part to the manuscript. The location of the revised parts is as follows: Page 10, line 356.

**Reviewer#2, SPECIFIC COMMENTS, Concern # 5 :**

-Page 12, 2nd paragraph: “: Could you comment on that and provide interpretation?

**Author response:** Thank you very much for your suggestion, we have added comments and explanations to this part. Comments and explanations added to the manuscript are listed below: The reason for the "sudden" changes in the sequence parameters can be analyzed from the earthquake sequence activity, which may be due to the occurrence of aftershocks of larger magnitude at the early stage after the main earthquake, thus the earthquake sequence has a large change in the decay rates of aftershock and the excitation degree of aftershock. However, this kind of sequence activity is only a representation analysis, and the adjustment of the stress field in the physical source area or the adjustment and change of the aftershock rupture mechanism needs to be further studied.

**Author action:** We have added comments and explanations to this part. The location of the revised parts is as follows: Page 12, line 400.

**Reviewer#2, SPECIFIC COMMENTS, Concern # 6 :**

- Figure 6 caption: Say the occurrence year better than the magnitude alone

**Author response:** Thank you very much for your suggestion, we have revised the title of Fig 5 to show the year of occurrence of the earthquake sequence. Specifically, we have revised the title of Fig 5 to "Retrospective forecasting test (using the earthquake sequence from February 12, 2014, in Hotan region as an example)".

**Author action:** We have revised the title of Fig 5 of the manuscript. The location of the revised parts is as follows: Page 15, figure 5, first line of title.

**Reviewer#2, SPECIFIC COMMENTS, Concern # 7 :**

-Page 13, 1st paragraph, 1st line: name the figure, give the figure number

**Author response:** Thank you very much for your suggestion! Based on your suggestion, we have given the number of this part of the figure as well as the title.

**Author action:** We have given the number of this part of the figure as well as the title. The location of the revised parts is as follows: Page 12, last paragraph, first line.

**Reviewer#2, SPECIFIC COMMENTS, Concern # 8 :**

-Page 14, 3rd paragraph: “… credible intervals” – which are these intervals? Hard to be shown in the graph. You need firstly to name the numbers and then to magnify the part where the values are almost all null.

**Author response:** Thank you very much for your suggestion! Based on your suggestion, we have described "credible intervals" in the title of Fig 5 with the following title: Fig 5 (top), the black dots indicate the number of earthquakes observed in each forecast period, the red solid line indicates the median number of earthquakes in the synthetic catalog for each forecast period, and the orange areas indicate the 95% forecast intervals for the number of earthquakes in each time. The extreme values for each interval are the 2.5% and 97.5% quantiles of the number of earthquakes in the synthetic catalog that make up the daily forecast. At the same time, we have added Fig 5 (below) giving the logarithm of the number of earthquakes, thus amplifying the almost blank part of this value.

**Author action:** We have shown "credible intervals" in the title of Fig 5 (top). At the same time, we have added Figure 5 (below). The location of the revised parts is as follows: Page 15, figure 5, title, fifth line.

**Reviewer#2, SPECIFIC COMMENTS, Concern # 9 :**

-Page 14, 3rd paragraph: “… of the strong aftershock” – when did they occur? How strong? How do you characterize then as “strong”?

**Author response:** Thank you very much for your suggestion! Based on your suggestion, we have added a detailed description of the "… of the strong aftershock” , here is Ms7.3 ( Hotan, February 12, 2014).

**Author action:** We have added a detailed description of the "… of the strong aftershock.” The location of the revised parts is as follows: Page 15, line 482.

**Reviewer#2, SPECIFIC COMMENTS, Concern # 10 :**

-Page 14, last paragraph: “… selection of the period …” – why July 2, 2023 specifically? You need to explain this choice.

**Author response:** Thank you very much for your suggestion! Based on your suggestion, we have added a reason for the "… selection of the period …". We have chosen the earthquake sequences from this region and time because of the large magnitude of the earthquakes and the long duration of the sequences, which therefore contain a large number of more complete earthquakes.

**Author action:** We have added a reason for the "… selection of the period …". The location of the revised parts is as follows: Page 16, line 489.

**Reviewer#2, SPECIFIC COMMENTS, Concern # 11:**

-Font size on the labels of Figure 7: Hard to be seen – please improve the figure

**Author response:** Thank you very much for your suggestion! Based on your suggestions, we have made revisions to Fig 6 in our manuscript, specifically revising the font size in the figure to become clearer.

**Author action:** We have made revisions to Fig 6 in our manuscript. The location of the revised parts is as follows: Page 17, figure 6.

**Reviewer#2, SPECIFIC COMMENTS, Concern # 12 :**

-Last paragraph: Please, better erase the: “of the paper”

**Author response:** We sincerely appreciate your careful reading. Based on your suggestions, we have deleted "of the paper" in the last paragraph.

**Author action:** We have deleted "of the paper" in the last paragraph. The location of the revised parts is as follows: Page 18, line 552.
