# Supplementary figures and images for: Correction: Spatio-temporal characterization of earthquake sequence parameters and forecasting of strong aftershocks in Xinjiang based on the ETAS model
Source: PLoS One. 2026 Apr 21;21(4):e0347626. doi: 10.1371/journal.pone.0347626 (PMC13098941; doi:10.1371/journal.pone.0347626)

**
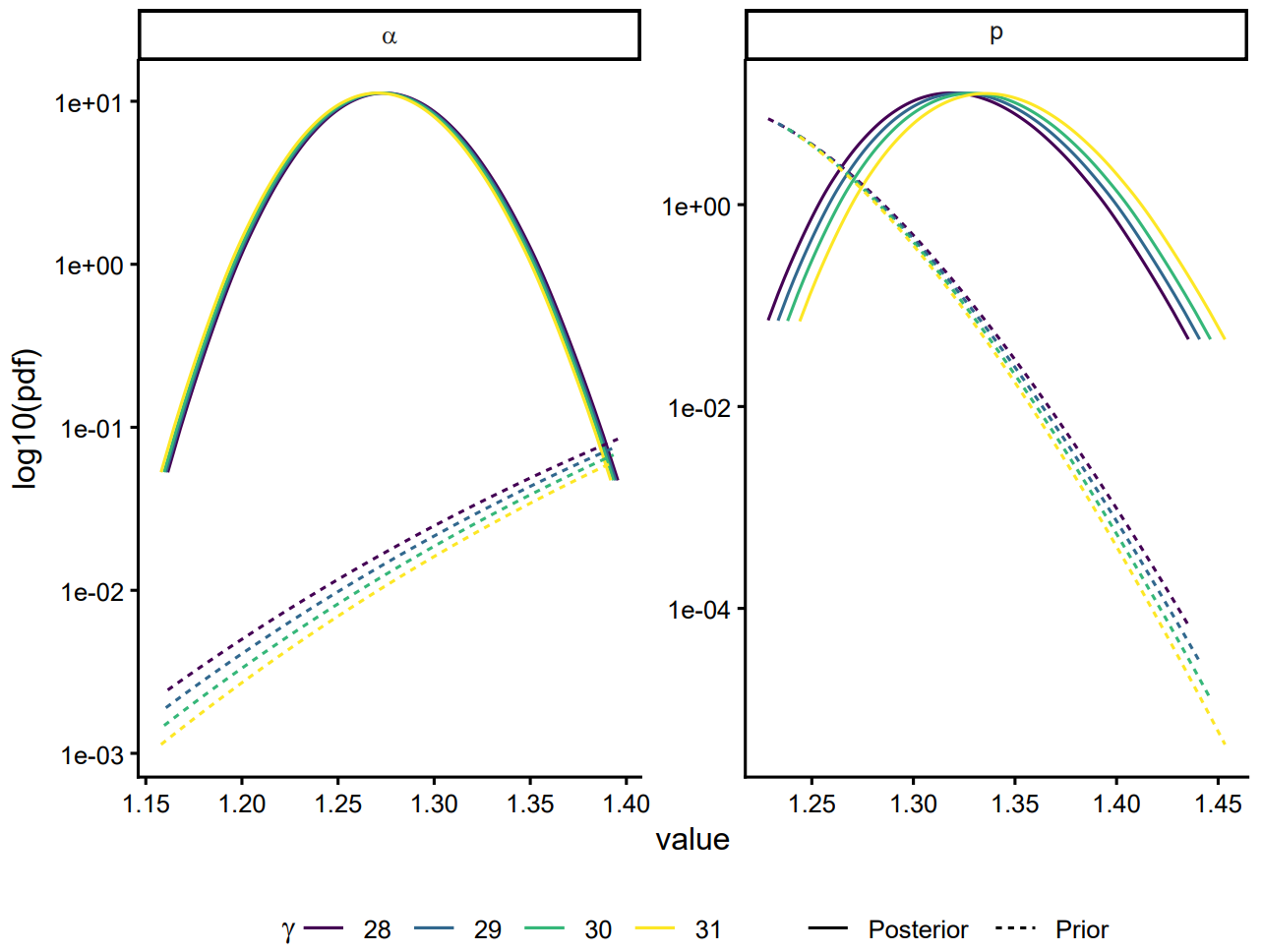
**

**S1 Fig. The posterior distribution of parameters α and p under different γ values.**

Supplement: S1 Fig — (DOCX) [file pone.0347626.s001.docx]

**
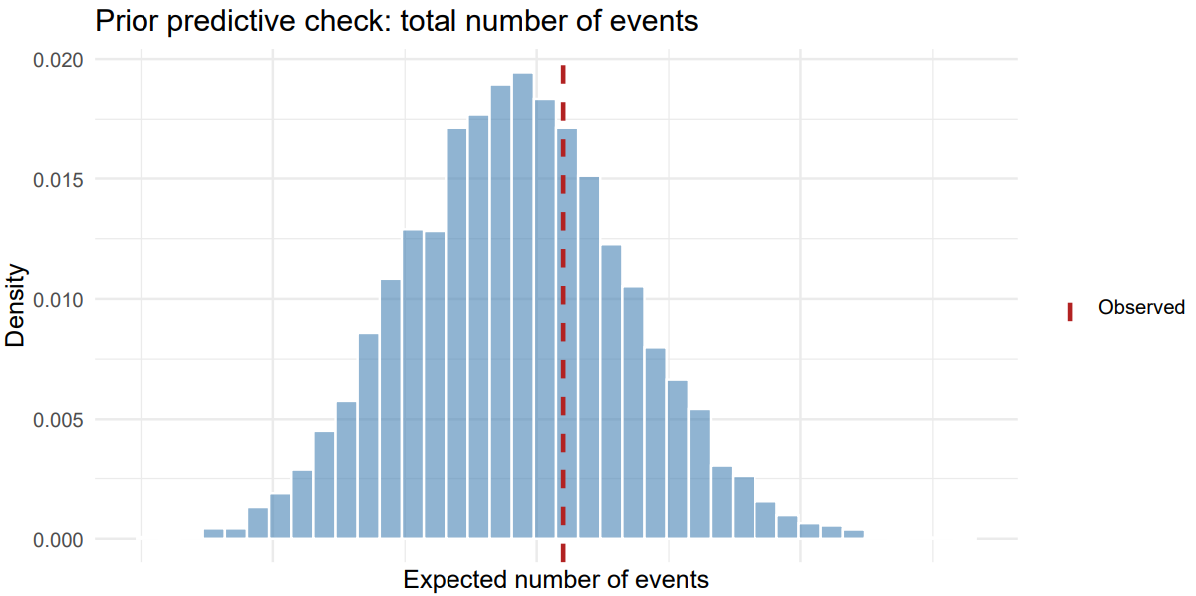
**

**S2 Fig. Priori predictive check for Hotan Ms7.3 and the priori setting results as shown in S1 Fig.**

Supplement: S2 Fig — (DOCX) [file pone.0347626.s002.docx]
