# Supplementary material for: Correction: Spatio-temporal characterization of earthquake sequence parameters and forecasting of strong aftershocks in Xinjiang based on the ETAS model
Source: PLoS One. 2026 Apr 21;21(4):e0347626. doi: 10.1371/journal.pone.0347626 (PMC13098941; doi:10.1371/journal.pone.0347626)
Supplement: S3 Fig — (DOCX) [file pone.0347626.s003.docx]

**
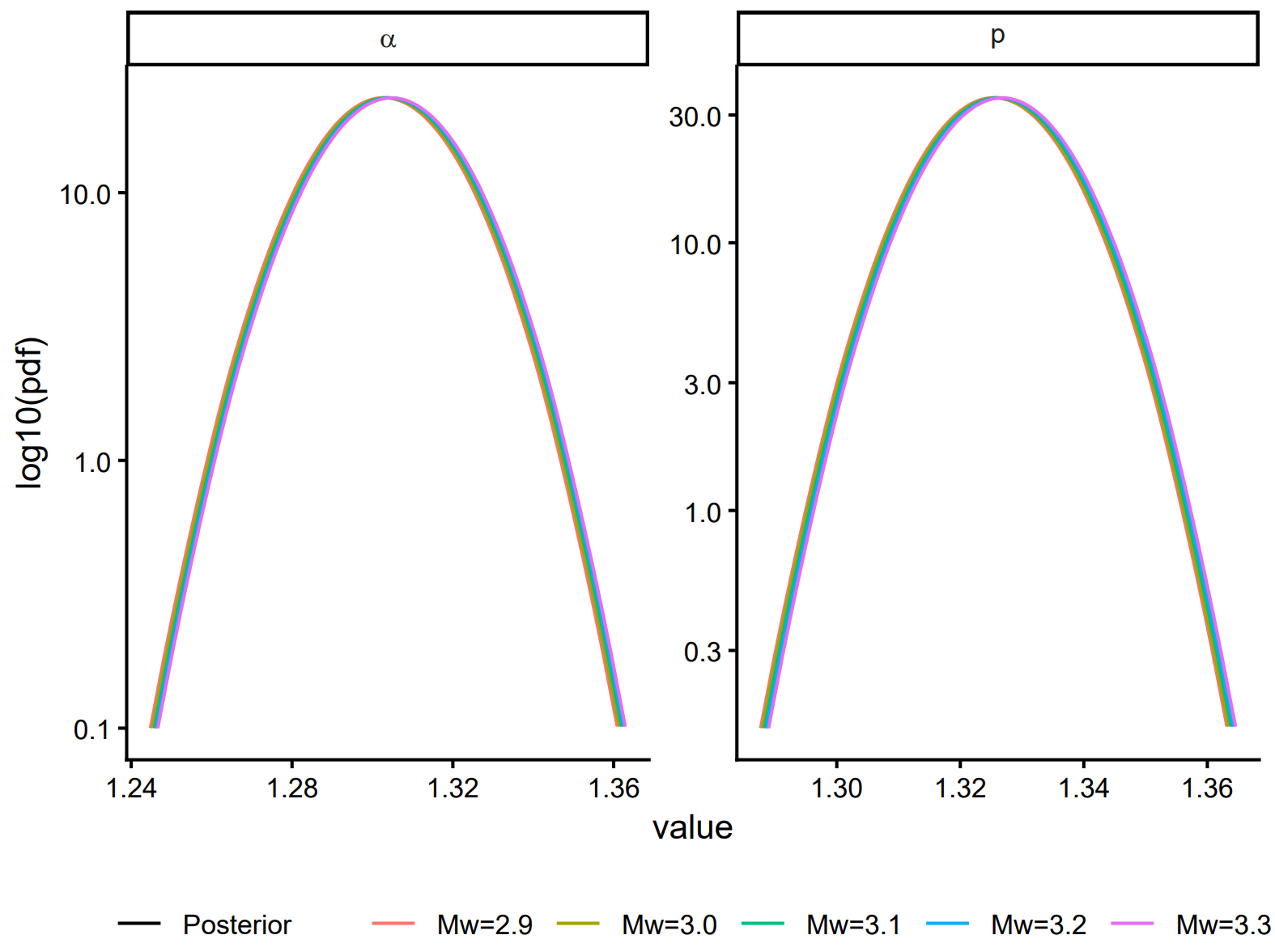
**

**S3 Fig. Posterior distributions of etas parameters α and p for the Hotan Ms7.3 sequence under varying integrity magnitudes (Mc) values.**
