# Supplementary material for: Correction: Spatio-temporal characterization of earthquake sequence parameters and forecasting of strong aftershocks in Xinjiang based on the ETAS model
Source: PLoS One. 2026 Apr 21;21(4):e0347626. doi: 10.1371/journal.pone.0347626 (PMC13098941; doi:10.1371/journal.pone.0347626)
Supplement: S4 Fig — (DOCX) [file pone.0347626.s004.docx]

**
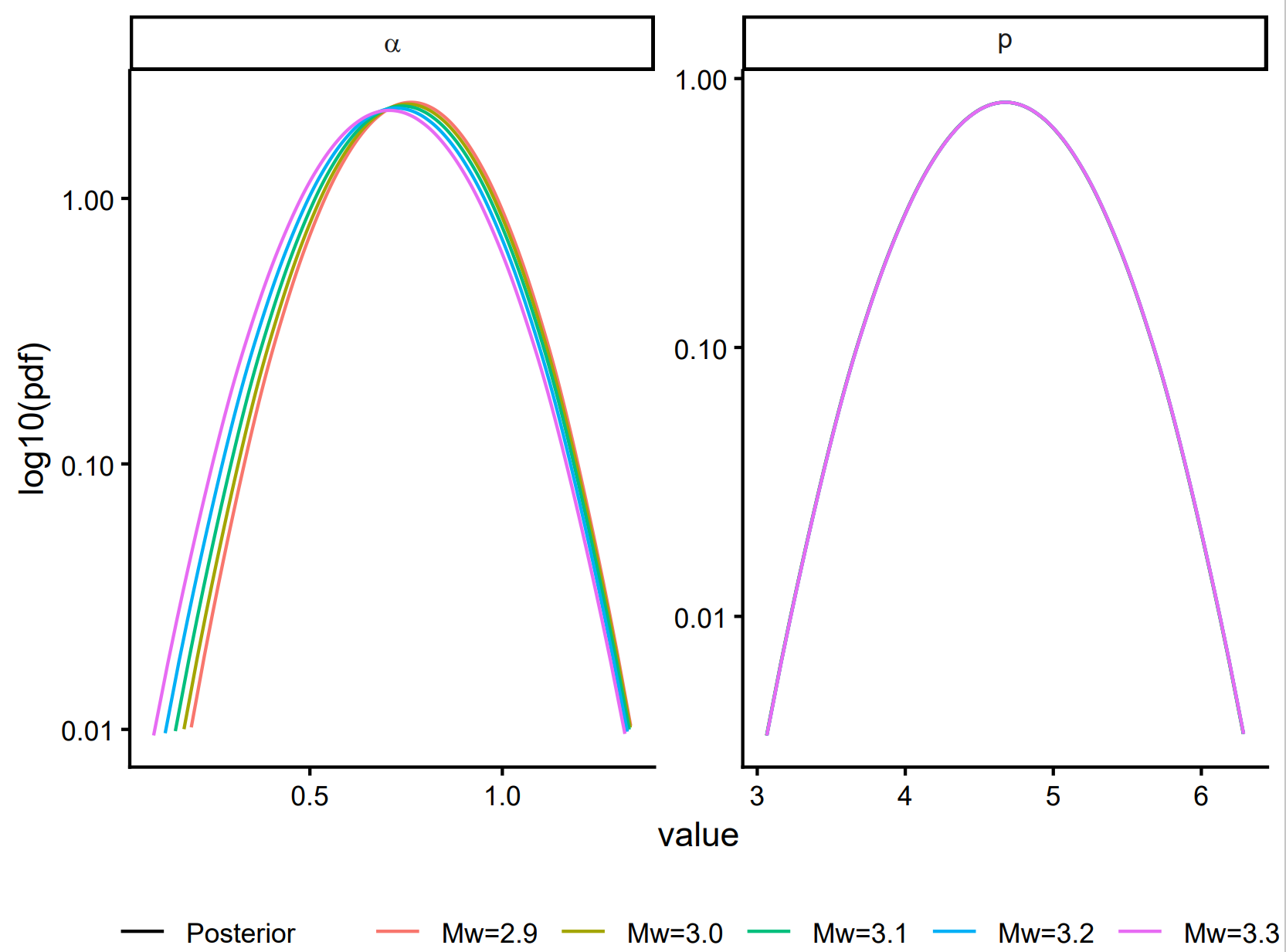
**

**S4 Fig. Posterior distributions of etas parameters α and p for the Kashgar Ms6.4 sequence under varying integrity magnitudes (Mc) values.**
