# Supplementary material for: Correction: Spatio-temporal characterization of earthquake sequence parameters and forecasting of strong aftershocks in Xinjiang based on the ETAS model
Source: PLoS One. 2026 Apr 21;21(4):e0347626. doi: 10.1371/journal.pone.0347626 (PMC13098941; doi:10.1371/journal.pone.0347626)
Supplement: S1 Table — (DOCX) [file pone.0347626.s005.docx]

| Mc | α (Mean) | α (95% CI) | p (Mean) | p (95% CI) |
| --- | --- | --- | --- | --- |
| 2.9 | 1.291 | [1.245, 1.337] | 1.332 | [1.306, 1.353] |
| 3.0 | 1.292 | [1.246, 1.338] | 1.332 | [1.307, 1.354] |
| 3.1 | 1.292 | [1.246, 1.339] | 1.332 | [1.308, 1.355] |
| 3.2 | 1.292 | [1.247, 1.340] | 1.332 | [1.308, 1.355] |
| 3.3 | 1.293 | [1.248, 1.341] | 1.333 | [1.309, 1.356] |

**S1 Table. The posterior mean and 95% confidence interval of parameters α and p in Hotan area under different integrity magnitudes (Mc).**
