# Supplementary material for: Correction: Spatio-temporal characterization of earthquake sequence parameters and forecasting of strong aftershocks in Xinjiang based on the ETAS model
Source: PLoS One. 2026 Apr 21;21(4):e0347626. doi: 10.1371/journal.pone.0347626 (PMC13098941; doi:10.1371/journal.pone.0347626)
Supplement: S2 Table — (DOCX) [file pone.0347626.s006.docx]

| Mc | α (Mean) | α (95% CI) | p (Mean) | p (95% CI) |
| --- | --- | --- | --- | --- |
| 2.9 | 0.635 | [0.455, 0.820] | 4.749 | [3.309, 5.200] |
| 3.0 | 0.618 | [0.443, 0.797] | 4.749 | [3.309, 5.200] |
| 3.1 | 0.602 | [0.435, 0.775] | 4.749 | [3.309, 5.200] |
| 3.2 | 0.588 | [0.425, 0.755] | 4.749 | [3.309, 5.200] |
| 3.3 | 0.575 | [0.412, 0.738] | 4.749 | [3.309, 5.200] |

**S2 Table. The posterior mean and 95% confidence interval of parameters α and p in Kashgar area under different integrity magnitudes (Mc).**
